# GO-SHIP Easy Ocean: Gridded ship-based hydrographic section of temperature, salinity, and dissolved oxygen

**DOI:** 10.1038/s41597-022-01212-w

**Published:** 2022-03-25

**Authors:** Katsuro Katsumata, Sarah G. Purkey, Rebecca Cowley, Bernadette M. Sloyan, Stephen C. Diggs, Thomas S. Moore, Lynne D. Talley, James H. Swift

**Affiliations:** 1 JAMSTEC, Yokosuka, 2370061 Japan; 2 Scripps Institution of Oceanography, La Jolla, California, 92093-0230 USA; 3 Oceans and Atmosphere, CSIRO, Hobart, Tasmania 7004 Australia; 4 CLIVAR and Carbon Hydrographic Data Office, Scripps Institution of Oceanography, La Jolla, California, 92093-0236 USA

**Keywords:** Physical oceanography, Physical oceanography, Marine chemistry, Marine biology

## Abstract

Despite technological advances over the last several decades, ship-based hydrography remains the only method for obtaining high-quality, high spatial and vertical resolution measurements of physical, chemical, and biological parameters over the full water column essential for physical, chemical, and biological oceanography and climate science. The Global Ocean Ship-based Hydrographic Investigations Program (GO-SHIP) coordinates a network of globally sustained hydrographic sections. These data provide a unique data set that spans four decades, comprised of more than 40 cross-ocean transects. The section data are, however, difficult to use owing to inhomogeneous format. The purpose of this new temperature, salinity, and dissolved oxygen data product is to combine, reformat and grid these data measured by Conductivity-Temperature-Depth-Oxygen (CTDO) profilers in order to facilitate their use by a wider audience. The product is machine readable and readily accessible by many existing visualisation and analysis software packages. The data processing can be repeated with modifications to suit various applications such as analysis of deep ocean, validation of numerical simulation, and calibration of autonomous platforms.

## Abstract (summary)

**Table:** 

Measurement(s)	temperature, salinity, and dissolved oxygen of sea water
Technology Type(s)	CTD (conductivity, temperature, depth profile) calibrated with bottle sampling

## Background & Summary

Owing to its volume and movement across seasonal to millennial time scales, the ocean is a key component in determining the climate state of the Earth System. High accuracy data are necessary to detect a statistically significant change to monitor the variability of the climate. For temperature (T), the standard requirement is 0.002 °C accuracy and 0.0005 °C precision^1^. This highly accurate measurement is often referred to as “climate quality”^2^. Similarly, climate quality salinity (S) data is required to study the freshwater budget of the Earth System. Technological advances in autonomous platforms have made near-global ocean observations more readily available^3,4^ for the upper 2000 m. Despite these advances, ship-based observations remain a key component of the ocean dataset enabling collection of contemporaneous ocean variables covering the full vertical extent of the water column. The accuracy of the global fleet of autonomous sensors depends on calibration against the ship-based hydrographic data. This is particularly the case for the nascent Deep Argo and Biogeochemical Argo programs. The continued collection of the highest quality data is coordinated by the Global Ship-Based Hydrographic Investigations Program (GO-SHIP, https://www.go-ship.org), which is a key component of Global Ocean Observing System, a UNESCO program under the Intergovernmental Oceanographic Commission. Highlights of the first decade of GO-SHIP achievements have been summarized in refs. ^2,5^. GO-SHIP data are publicly available from CCHDO (https://cchdo.ucsd.edu), the central data archive for GO-SHIP.

**Fig. 1:**
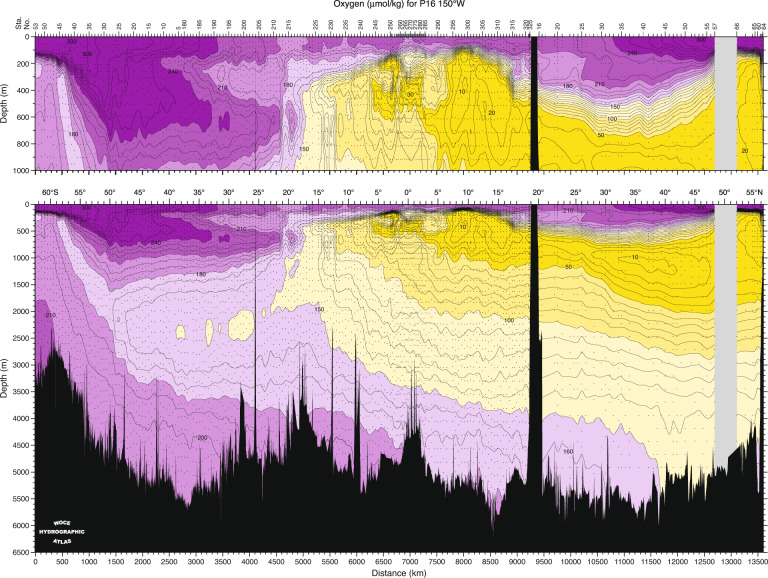
Dissolved oxygen concentration along the Pacific hydrographic section P16, nominally along 150°W from the World Ocean Circulation Experiment (WOCE) that preceded and set the section locations and protocols continued in GO-SHIP. The distribution demonstrates the meridional overturning circulation in the Pacific; sinking of a newly-ventilated water mass with high oxygen concentration around Antarctica, followed by northward penetration along the bottom and gradual lightening, losing oxygen by biological activity, and a southward return path at mid-depth. A cell of near surface overturning circulation can also be discerned. While this particular figure was produced with rosette sampled oxygens (small dots), it can equally be created from the CTDO profiles at the same stations, available through GO-SHIP Easy Ocean. Reproduced from Talley (2007)^11^.

The GO-SHIP archive contains a rich ocean data record. For example, a meridional section of dissolved oxygen (Fig. 1), from the predecessor World Ocean Circulation Experiment (WOCE), is often used in classrooms to demonstrate the meridional overturning of the ocean. However, the data structure is complicated and can challenge new users or those not familiar with irregular data. First, data are stored not by section, but by voyage, and often multiple legs constitute a section. Logistical capacity of research ships limits duration or distance between port calls, resulting in reference sections often being divided into two or more legs. Station spacing is inherently uneven because of the requirement to resolve boundary currents, topographically steered flows etc. and can also vary from one occupation to another depending on available shiptime. Altered station locations across multiple occupations of a given section also result from voyage delays, including mechanical issues, weather, and medical evacuations, often resulting in wider station spacing, and missing or incomplete stations. Hence, when a user wishes to compare multiple occupations of one section, gridding is usually required because station locations are usually not consistent across different occupations of a section.

Another common impediment to easy use of GO-SHIP data is inconsistent data formatting. While all observations are machine readable, data processing workflows can fail due to minor but numerous differences across the data from different voyages. For example, bottom depth may be recorded in the summary file in some cases and in the header of the data files in others. Further, the precise combination of sensors often differs from voyage to voyage leading to a multitude of differences in data file structure. Finally, comparison between voyages across many decades can be problematic, depending on intended use of the data, given that the accuracy of ocean measurements has improved over the last forty years. For salinity measurement, it is well known that the Standard Sea Water used for calibration has batch-to-batch biases^6,7^. These biases can be minimised by applying the offsets determined in shore-based laboratories^8^. These offsets are of order of 10^−3^ in g/kg, equal in size to natural salinity changes reported in deep oceans^9^.

Here we introduce the GO-SHIP Easy Ocean data product to enable wider use of this unique observational data set. GO-SHIP Easy Ocean provides access to a gridded, machine-readable hydrographic ocean data set, readily available for both data analysts and modellers. In this first version of the product, GO-SHIP and WOCE Conductivity-Temperature-Depth-Oxygen (CTDO) data have been downloaded from CCHDO and processed into a more user-friendly database. Table 1 lists typical visualisation and analysis software and the pertinent GO-SHIP Easy Ocean format. The listed JAVA Ocean Atlas (https://joa.ucsd.edu) also includes complementary quality-controlled bottle data. In addition, we provide access to software that enables users to tailor the data processing and/or interpolation methods to their specific purposes.

**Table 1:** Some popular data visualisation software packages with the appropriate compatible GO-SHIP Easy Ocean format file.

Application	Reported	Gridded
**Matlab**	reported/P16/p16.mat	gridded/P16/p16.mat
**Ocean Data View (**http://odv.awi.de**)**	reported/P16/p16_2015_ct1.zip	—
**JAVA Ocean Atlas (**http://joa.ucsd.edu**)**	reported/P16/p16_2015_ct1.zip	—
**GrADS (**http://cola.gmu.edu/grads**)**	—	gridded/P16/p16.bin.ctl
**binary**	—	gridded/P16/p16.bin
**ASCII** **GMT (**https://www.generic-mapping-tools.org**)**	reported/P16/p16_2015_ct1.zip	gridded/P16/p16_2015.xyz.gz
**NetCDF**	(work in progress)	gridded/P16/p16_2015.nc

Example is shown for the P16 Section in 2014–2015, but is applicable to all sections.

## Methods

**Fig. 2:**
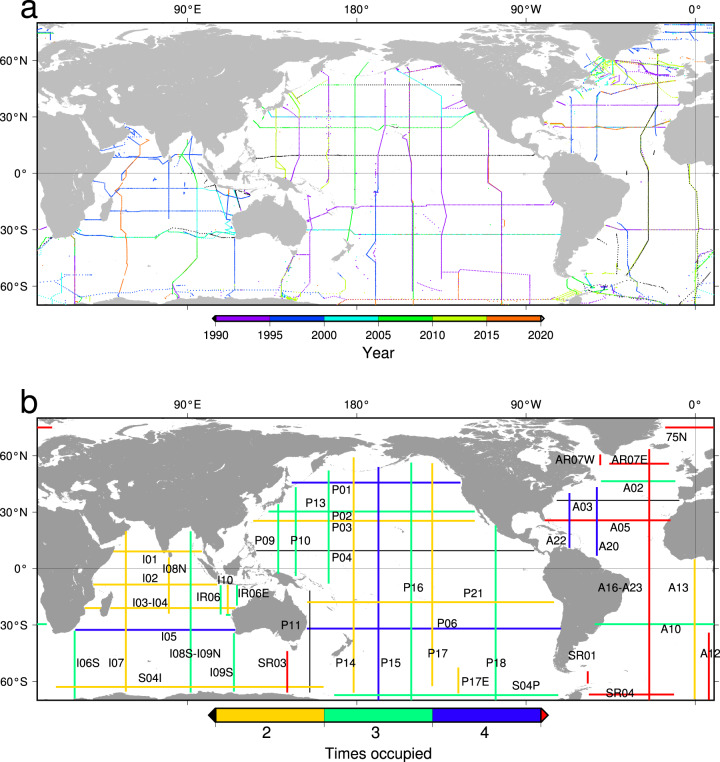
(**a**) A map of hydrographic stations included in the section data used to produce the gridded GO-SHIP Easy Ocean data product. The colour shows the year of occupation. Note many stations overlap. All stations included in the raw data are shown, some of which are not included in the final gridded product (Fig. 2b). (**b**) Approximate locations of GO-SHIP Easy Ocean gridded sections with section names. The colour shows how many times (at least partially) the sections have been occupied as of September 2021.

The original Conductivity-Temperature-Depth-Oxygen (CTDO) data were downloaded from CCHDO (Fig. 2a). For each section, the stations were compared with those used in the WOCE Atlas (http://woceatlas.ucsd.edu/), vertical section data labelled “The Best” in the JAVA Ocean Atlas (https://joa.ucsd.edu), and ref. ^9^.

The *station list*s were edited to include only those stations that correspond to the occupation of a section, as many voyages included additional, unassociated stations. A hydrographic section is a set of stations placed along a line connecting landmasses or other sections, mostly defined in WOCE. A *station list* is an ASCII file with each line consisting of latitude and longitude, date and time, depth, station number and cast number used in the original dataset. Those stations not to be included in the *reported* or *gridded* products (explained below) are commented out. This selection has been manually performed by the authors but a user can make a different choice by commenting in or out stations.

The choice of the stations included in any given section is subjective, based on the authors’ best scientific judgement. There was no standard against which station selection has been made for the product. (Note that the online WOCE Indian and Pacific atlases^10,11^ include station lists used for sections, and that station lists for all WOCE atlases can be inferred from the vertical sections therein.) Similarly, it was a subjective decision which cruises to combine to form one ‘occupation’ when multiple cruises were conducted over an interval of a few years. We did not attempt to produce a standard or definitive product, but encourage the user to build their own product best fit for their purpose. The GO-SHIP Easy Ocean distributed product is one incarnation of what is possible, built loosely on scientific understanding of regional spatial variability in the ocean to determine when a station is close enough to be considered on the original line. It should also be noted that sections often have multiple section designators (e.g. A01E and AR07E). Correspondence between cruises and sections are listed in the online table for the Standard Sea Water batch offset (SaltBatchOffset/README.md) in the source code repository (see Code Availability below).

Cruise documents distributed with the datasets from CCHDO were consulted to determine the Standard Sea Water batch correction and the batch-to-batch offset was applied to the salinity data^7,8^. The batch correction was not applied when the batch number was not known. Out of 245 voyages, we have found Standard Sea Water batch numbers for 188, either in the cruise documentation or through personal communications with the Principal Investigators. A user can remove the correction by reproducing the product without the batch correction option (see detail in Procedure.md file in the software repository).

Once the station list and batch corrections were determined, the downloaded hydrographic data were converted to the formats explained in the next section. Due to slight differences in file format, modifications to software were often required to complete the process. These interventions were recorded in the README.md files in the repository. The use of a station list provides a straightforward way to customise portions of the processing pipeline (e.g. gridding procedure) and re-process the results for future purposes specific to user needs. The software is implemented using MATLAB with the TEOS-10 library^12^. The locations of the processed sections are shown in Fig. 2a and the approximate locations of gridded sections are shown in Fig. 2b.

## Data Records

GO-SHIP Easy Ocean^13^ sections are available from a dedicated site at CCHDO (10.7942/GOSHIP-EasyOcean) and no registration is needed to download the data set. Un-interpolated (voyage *reported*) and interpolated (horizontally and vertically *gridded*) products are available. As of 2021, the product consists of 46 CTDO sections with a total of 201 occupations. As new occupations are completed these observations will be added to GO-SHIP Easy Ocean in the future.

For both the *reported* and *gridded* formats, six quantities (one, pressure, used as vertical axis) are recorded (Table 2): *in situ* temperature in ITS-90 scale, *in situ* salinity in PSS-78 scale, dissolved oxygen concentration in μmol/kg (absent if source CTD data do not provide dissolved oxygen data), Conservative Temperature in °C, and Absolute Salinity in g/kg.

**Table 2:** Available quantity parameter names in reported files.

Quantity	Column Header	Units
**Corrected PRESSURE**	CTDPRS	DBAR (deci bar)
**Corrected TEMPERATURE**	CTDTMP	ITS-90 (International Temperature Scale 90)
**Corrected** ***in situ*** **SALINITY**	CTDSAL	PSS-78 (Practical Salinity Scale 78)
**Dissolved OXYGEN concentration**	CTDOXY	μmol/kg
**Conservative Temperature**	CTDCT	°C
**Absolute Salinity**	CTDSA	g/kg

### Reported Data

Uninterpolated station data, as reported by each voyage, are called *reported* data. A zipped archive holds all stations used to create the gridded section. Each file is in CSV (comma separated values) text (UTF-8 encoding) and thus compatible with widely-used spreadsheet software as well as text editors. The files follow the WOCE Hydrographic Program Exchange format (https://cchdo.ucsd.edu/formats), which major ocean data visualisation tools such as JAVA Ocean Atlas and Ocean Data View can handle. MATLAB readable binary data are also provided. The NetCDF version of the reported data is in preparation.

### Gridded Sections

**Fig. 3:**
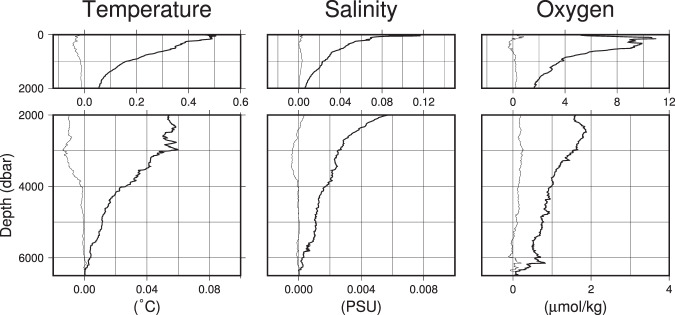
Errors introduced in the gridding procedure. The thin lines show the average of the errors (biases) and the thick lines show the standard deviation of the errors estimated at each depth. The errors for Conservative Temperature and Absolute Salinity are not distinguishable from those for temperature and salinity, respectively, and not plotted.

Interpolated (horizontally and vertically) section data are here called *gridded* data. The meridional or zonal station data are interpolated to a standard 0.1° horizontal grid. Vertically, the data were interpolated to a 10 dbar grid with a Gaussian filter (see vinterp_gauss.m in the code). We used an objective mapping method by Roemmich^10^ (hinterp_objmap.m). The method has been successfully applied in previous vertical sections^11,14^. The mapping is performed in two steps; the first for the large scale field with a horizontal scale of 40 station spacing (approximately 2200 km, signal-to-noise ratio 0.1) and the second for the eddy field with a scale of two station spacing (approximately 110 km, signal-to-noise ratio 0.3). The distance of 40 stations varies less than 40% among occupations and the gridding is not sensitive to the choice of the correlation length (doubling the correlation length yielded root-mean-square differences an order smaller than the interpolation errors shown in Fig. 3). The horizontal interpolation was performed within a *basin*, where two stations separated more than two degrees in longitude/latitude were deemed as in different *basins*. The user can not only change this criterion but also substitute other interpolation methods if needed. Bottom depths are estimated by linearly interpolating the depths at the neighbouring stations. The ASCII text output has seven columns; latitude or longitude, pressure, temperature, salinity, dissolved oxygen concentration, Conservative Temperature, and Absolute Salinity (Conservative Temperature and Absolute Salinity are the new standard variables^12^ for heat and salt adopted by International Oceanographic Commission. See http://www.teos-10.org/ for detail). If the source CTDO data do not provide the dissolved oxygen profile, this is indicated with missing values. Binary output in IEEE754 format is also provided where the first datum in the IEEE output is the southern-most/western-most shallowest temperature. The second is the shallowest temperature from the next horizontal grid point. After temperature, salinity data are stored in the same spatial arrangement as temperature.

MATLAB readable output and CF compliant (CF-1.7, ACDD-1.3) NetCDF binary files are also provided. The NetCDF files are dimensioned by gridded_section (an integer indicating the gridded section number), longitude, latitude and depth. In addition to temperature, salinity, dissolved oxygen concentration, Conservative Temperature, and Absolute Salinity variables, we have included a time variable by assigning a date to each gridded data point equal to the collection date of the closest station, rounded to the day. Global attributes in the files include information on the CCHDO voyage identifiers used, data source links, and years of the voyages.

There is a caveat for the use of the gridded data. We chose longitude or latitude for the horizontal coordinate of the gridded product. If the distance between the northernmost and southernmost stations is larger than the distance between the easternmost and westernmost stations, the section is meridional, and vice versa for zonal. When the section has oblique trajectory (e.g. southeastward ship track), this information is lost in the gridded product. Sometimes the station location can be hundreds of kilometers off from the extension of the main meridional/zonal sections. Extra care is required for interpretation of data from these stations. Those users interested in data intercomparison or inventory calculations should use the reported format.

## Technical Validation

The accuracy and precision of the *reported* data follows the GO-SHIP standard^1^; accuracy = 0.002 ^o^C and precision = 0.0005 ^o^C for temperature, accuracy = 0.002 g/kg and precision = 0.001 g/kg for Absolute Salinity under TEOS-10^12^, accuracy = 3 dbar and precision = 0.5 dbar for pressure, and accurarcy = precision = 1% for dissolved oxygen. Data possibly not meeting the standard are indicated by the quality flag. No additional quality control is performed other than data selection through the quality flag provided in the source data. For the distributed product, only those data flagged “acceptable” (flag = 2) were included. In addition, a few obvious errors have been manually removed and tabulated in README.md file of the software repository.

In addition to these measurement errors, the gridding process introduces errors to the *gridded* data product. These errors were estimated by randomly removing one station in each *basin* (as defined in the previous section). The interpolated values at the grid point nearest to the removed station were compared with the observed values at the station and the difference was regarded as the error. The procedure was repeated ten times and the interpolation error was obtained at 2770 stations. The depth profiles of the interpolation error for temperature, salinity, and dissolved oxygen are shown in Fig. 3. The interpolation errors for depths <1000 dbar is largest (0.5 °C, 0.1, and 10 μmol/kg, respectively). In deep waters (>4000 dbar), the errors are of the same order as the natural decadal variability (0.01 °C, 0.002, and 1 μmol/kg, respectively) and require careful treatment when discussing the decadal variability (e.g. Fig. 5 below). The average of errors (i.e. biases) were likely caused by eddies not resolved by interpolation.

## Usage Notes

The GO-SHIP Easy Ocean product provides access to an observational-based climate quality gridded dataset. This product can be used for many purposes, including but not limited to, validation of model simulations, comparison to process studies, and quantification of decadal ocean change.

**Fig. 4:**
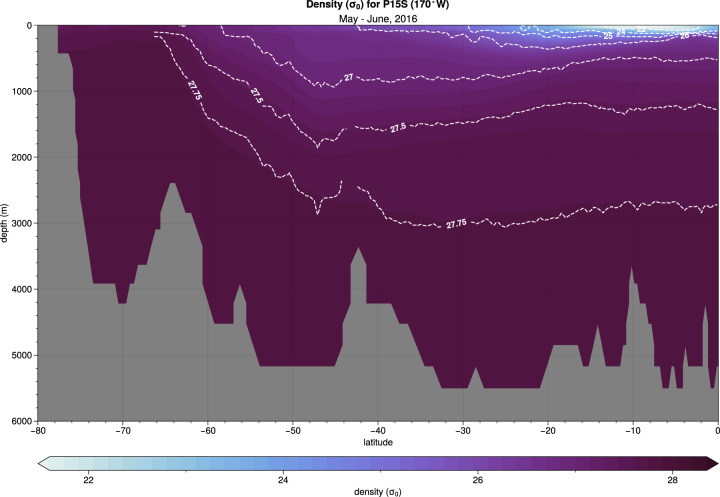
Pacific Ocean density (${\sigma }_{0}$) along 170°W (P15 between 70°S-0°S, see Fig. 5) observed in May and June 2016 from the GO-SHIP Easy Ocean gridded data product (white contours) overlaid on density (${\sigma }_{0}$) from the CAFE60^15^ reanalysis product averaged over the same period. Colormap is from ref. ^23^.

Here we show a comparison of the Pacific Ocean density structure along 170°W (GO-SHIP P15 section) observed in May and June 2016 from the GO-SHIP Easy Ocean gridded data product (white contours) overlaid on density from the CAFE60 reanalysis product^15^ averaged over the same period (Fig. 4). The CAFE60 product, derived from a coupled general circulation model with data assimilation implemented via an ensemble transform Kalman filter, has the same density structure as observed with outcropping of denser water masses in a southward direction. Note that the P15 data were assimilated by CAFE60 only indirectly after strong smoothing so this direct comparison serves as a useful check.

**Fig. 5:**
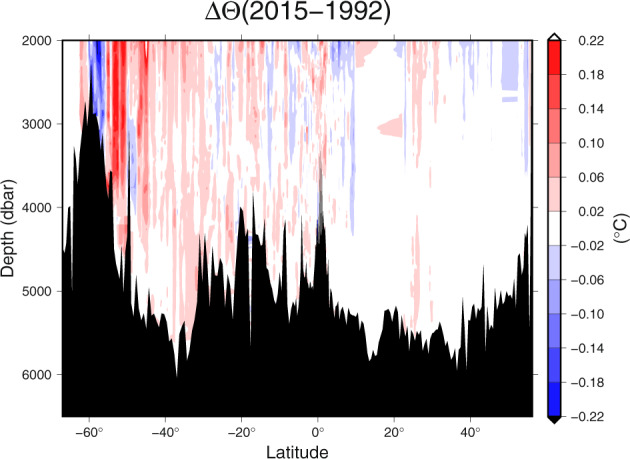
The difference in the observed Pacific Ocean Conservative Temperature (°C) along 150°W (P16) between 1992 and 2015 using the GO-SHIP Easy Ocean gridded data product.

Using the gridded product, it is a relatively simple task to difference the data between different occupations. A simple script GMTplotDiff.sh (included in our software repository with usage description within the script) using GMT^16^ enables one to produce differences in time along sections for scientific analyses^9,17–19^. Here we show the difference in temperature observed on the P16 section (150°W) between the occupation in 2015 and 1992 (Fig. 5). Warming south of 30 °S, with magnitude larger than 0.2 °C (greater than the measurement accuracy but comparable to the interpolation error) is obvious. This warming has been reported^19,20^.

The same method of using *station list*s (defined in Methods) to produce gridded sections can be applied to other quantities such as carbon parameters and CFCs, which are also available through CCHDO and have been further quality-controlled and compiled in GLODAPv2^21^ (https://www.ncei.noaa.gov/access/ocean-carbon-data-system/oceans/) as well as in section compilations in JAVA Ocean Atlas. This initial release of GO-SHIP Easy Ocean includes temperature, salinity, and dissolved oxygen data from Conductivity-Temperature-Depth-Oxygen profiles, but the product will include other properties such as nutrients and carbon parameters in future releases.

## Data Availability

Matlab computer software used to produce GO-SHIP Easy Ocean product is available from the GO-SHIP Easy Ocean GitHub repository (https://github.com/kkats/GO-SHIP-Easy-Ocean). The present paper is based on version 1.4 10.5281/zenodo.5527383). A user can substitute their preferred stations and gridding method by implementing their workflow in MATLAB and re-running the batch file. The guidelines to reproduce the work are found in the Procedure.md file in the software repository.
